# P-287. Risk factors for Pseudomonas aeruginosa in ICU-Related Respiratory Infections. An analysis from the European Network for ICU-Related Respiratory Infections (ENIRRIs) study

**DOI:** 10.1093/ofid/ofae631.490

**Published:** 2025-01-29

**Authors:** Cristian C Serrano-Mayorga, Juan Olivella-Gomez, Antoni Torres, Ignacio Martin-Loeches, Luis Felipe F Reyes

**Affiliations:** Universidad de La Sabana, Chía, Cundinamarca, Colombia; Clínica Universidad de La Sabana, Bogotá, Distrito Capital de Bogota, Colombia; Hospital Clinic of Barcelona, Barcelona, Catalonia, Spain; Saint James University Hospital, Dublin, Dublin, Ireland; Universidad de La Sabana, Chía, Cundinamarca, Colombia

## Abstract

**Background:**

Nosocomial low respiratory infections (nLRTIs) represent a significant complication prevalent among patients who require admission to the intensive care unit (ICU). Gram-negative microorganisms are related to nLRTIs, such as ventilator-associated pneumonia (VAP), ventilator-associated tracheobronchitis (VAT), ICU-acquired pneumonia (ICU-HAP), and hospital-acquired pneumonia (HAP). Pseudomonas aeruginosa (PA) is frequently identified among nLRTIs; however, not all patients with nLRTI may require antipseudomonal treatment and this is not clear in the literature. This study aims to describe the characteristics and principal outcomes of patients with confirmed PA nLRTI.

**Table 1.**
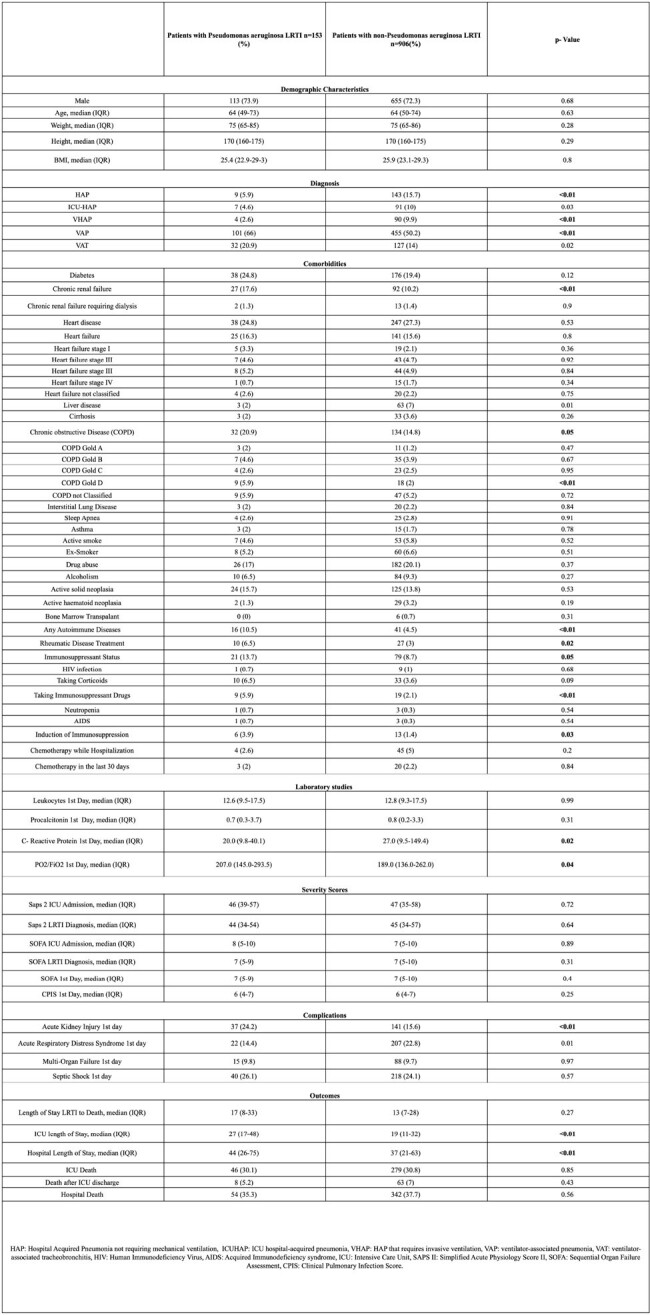
Characteristics of patients with Pseudomonas aeruginosa identification Vs Non-Pseudomonas aeruginosa identification.

**Methods:**

This prospective cohort was conducted in 12 countries over two continents from 9th May 2016 until 16th August 2019. Characteristics and outcomes of nLRTI’s patients admitted to the ICU were collected. All patients were tested to find microbiological-causing agents. Patients with confirmed isolation for PA were compared to patients with non-PA isolation. The Mam Whitney and Chi-squared were used to compare the group's proportions depending on their distribution.

**Table 2.**
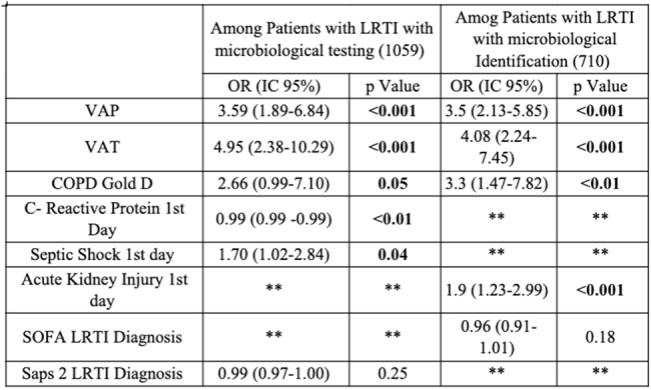
Bivariate analysis for risk factors associated with Pseudomonas aeruginosa

**Results:**

1059 patients with nLRTIs who underwent testing for microbiological diagnosis were included, 14.4% patients had confirmed infection by PA. 74% were male with a median age of 64 (49-73) years old. The principal diagnosis among patients with PA was VAP. The principal comorbidities were diabetes and heart disease, followed by COPD. The most frequent complication was septic shock, followed by acute kidney injury. There were differences among the comorbidities, complications and outcomes among the groups (Table 1). However, there were no differences in mortality (Table 1 - Figure 1). VAT and VAP diagnosis, COPD Gold D and AKI on the 1st day were associated with a high risk of infection by PA (Table 2).

**Figure 1.**
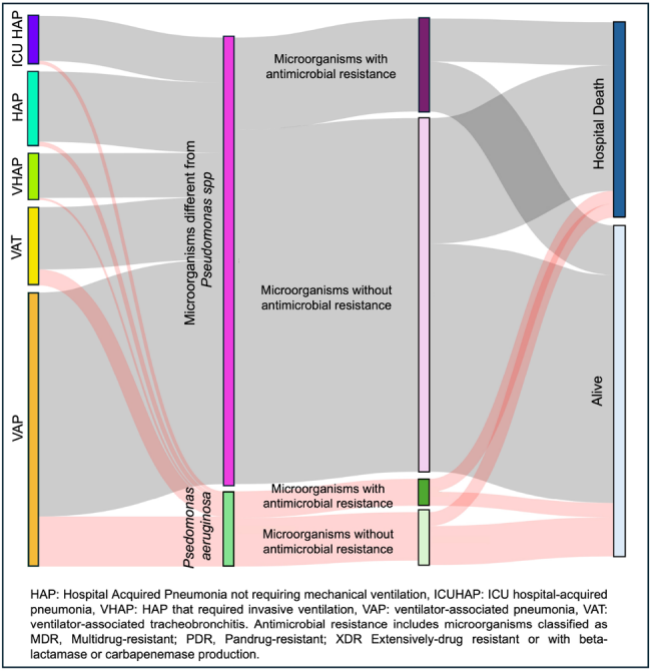
Sankey diagram showing mortality outcome. Pseudomonas aeruginosa Vs. Non-Pseudomonas aeruginosa LRTI-diagnosed patients who were admitted to the ICU.

**Conclusion:**

This multi-country study found some important risk factors associated with PA in nLRTI patients. Differences among groups were notorious; diabetes and COPD comorbidities are more frequent among PA nLRTI patients. Finally, PA infection increases the length of stay of patients. Further studies are needed to confirm these findings.

**Disclosures:**

**All Authors**: No reported disclosures

